# Efficacy of single versus four repeated doses of praziquantel against *Schistosoma mansoni* infection in school-aged children from Côte d'Ivoire based on Kato-Katz and POC-CCA: An open-label, randomised controlled trial (RePST)

**DOI:** 10.1371/journal.pntd.0008189

**Published:** 2020-03-20

**Authors:** Pytsje T. Hoekstra, Miriam Casacuberta-Partal, Lisette van Lieshout, Paul L. A. M. Corstjens, Roula Tsonaka, Rufin K. Assaré, Kigbafori D. Silué, Aboulaye Meité, Eliézer K. N’Goran, Yves K. N’Gbesso, Abena S. Amoah, Meta Roestenberg, Stefanie Knopp, Jürg Utzinger, Jean T. Coulibaly, Govert J. van Dam

**Affiliations:** 1 Department of Parasitology, Leiden University Medical Center, Leiden, the Netherlands; 2 Department of Cell and Chemical Biology, Leiden University Medical Center, Leiden, the Netherlands; 3 Department of Biomedical Data Sciences, Leiden University Medical Center, Leiden, the Netherlands; 4 Centre Suisse de Recherches Scientifiques en Côte d’Ivoire, Abidjan, Côte d’Ivoire; 5 Unité de Formation et de Recherche Biosciences, Université Félix Houphouët-Boigny, Abidjan, Côte d’Ivoire; 6 Swiss Tropical and Public Health Institute, Basel, Switzerland; 7 University of Basel, Basel, Switzerland; 8 Programme National de Lutte contre les Maladies Tropicales Négligées à Chimiothérapie Préventive, Ministère de la Santé et de l’Hygiène Publique, Abidjan, Côte d’Ivoire; 9 Département d’Agboville, Centre de Santé Urbain d’Azaguié, Azaguié, Côte d’Ivoire; 10 Department of Population Health, Faculty of Epidemiology and Population Health, London School of Hygiene and Tropical Medicine, London, United Kingdom; 11 Malawi Epidemiology and Intervention Research Unit, Chilumba, Karonge District, Malawi; 12 Department of Infectious Diseases, Leiden University Medical Center, Leiden, the Netherlands; Johns Hopkins University, UNITED STATES

## Abstract

**Background:**

Preventive chemotherapy with praziquantel (PZQ) is the cornerstone of schistosomiasis control. However, a single dose of PZQ (40 mg/kg) does not cure all infections. Repeated doses of PZQ at short intervals might increase efficacy in terms of cure rate (CR) and intensity reduction rate (IRR). Here, we determined the efficacy of a single versus four repeated treatments with PZQ on *Schistosoma mansoni* infection in school-aged children from Côte d’Ivoire, using two different diagnostic tests.

**Methods:**

An open-label, randomized controlled trial was conducted from October 2018 to January 2019. School-aged children with a confirmed *S*. *mansoni* infection based on Kato-Katz (KK) and point-of-care circulating cathodic antigen (POC-CCA) urine cassette test were randomly assigned to receive either a single or four repeated doses of PZQ, administered at two-week intervals. The primary outcome was the difference in CR between the two treatment arms, measured by triplicate KK thick smears 10 weeks after the first treatment. Secondary outcomes included CR estimated by POC-CCA, IRR by KK and POC-CCA, and safety of repeated PZQ administration.

**Principal findings:**

During baseline screening, 1,022 children were assessed for eligibility of whom 153 (15%) had a detectable *S*. *mansoni* infection, and hence, were randomized to the standard treatment group (N = 70) and the intense treatment group (N = 83). Based on KK, the CR was 42% (95% confidence interval (CI) 31–52%) in the standard treatment group and 86% (95% CI 75–92%) in the intense treatment group. Observed IRR was 72% (95% CI 55–83%) in the standard treatment group and 95% (95% CI 85–98%) in the intense treatment group. The CR estimated by POC-CCA was 18% (95% CI 11–27%) and 36% (95% CI 26–46%) in the standard and intense treatment group, respectively. Repeated PZQ treatment did not result in a higher number of adverse events.

**Conclusion/significance:**

The observed CR using KK was significantly higher after four repeated treatments compared to a single treatment, without an increase in adverse events. Using POC-CCA, the observed CR was significantly lower than measured by KK, indicating that PZQ may be considerably less efficacious as concluded by KK. Our findings highlight the need for reliable and more accurate diagnostic tools, which are essential for monitoring treatment efficacy, identifying changes in transmission, and accurately quantifying the intensity of infection in distinct populations. In addition, the higher CR in the intense treatment group suggests that more focused and intense PZQ treatment can help to advance schistosomiasis control.

**Trial registration:**

www.clinicaltrials.gov NCT02868385.

## Author summary

The previously established efficacy of the widely used drug praziquantel (PZQ) against schistosomiasis might have been overestimated due to the use of inaccurate diagnostic methods. Repeated PZQ treatment at short intervals in areas with ongoing transmission could more effectively target non-susceptible schistosomula as they will have matured into drug susceptible worms within a few weeks. In the current study, we aimed to determine the cure rate (CR) of repeated PZQ, measured by the Kato-Katz (KK) technique and the point-of-care circulating cathodic antigen (POC-CCA) test, respectively. An open-label, randomized controlled trial was conducted assigning 153 school-aged children with a confirmed *Schistosoma mansoni* infection to two groups, one receiving a single PZQ treatment, while the second group received four repeated PZQ treatments, given at two-week intervals. Based on the KK test, the CR was significantly higher after four repeated treatments compared to a single treatment. When using POC-CCA, a diagnostic method that has not been utilized before in studies assessing the efficacy of four repeated PZQ treatments, the CR was much lower, even after four repeated PZQ treatments. Our results indicate that worms are still present after multiple PZQ treatments and that PZQ might be less efficacious than previously published.

## Introduction

Schistosomiasis remains a public health problem in different parts of the world with an estimated 779 million people at risk of infection and more than 250 million people infected [1, 2]. The disease is caused by parasitic blood flukes of the genus *Schistosoma*. The three most important species are *S*. *japonicum* and *S*. *mansoni* (causing intestinal schistosomiasis) and *S*. *haematobium* (causing urogenital schistosomiasis) [3, 4]. To control schistosomiasis, health authorities rely on preventive chemotherapy, that is the large-scale administration of the anthelmintic drug praziquantel (PZQ) to at risk populations without prior diagnosis [5]. This strategy has been successful in reducing the prevalence and, most importantly, the intensity of infection, and thereby controlling morbidity [6, 7]. The burden of schistosomiasis is greatest in school-aged children, generally presenting the highest prevalence and intensity of infection [8]. School-aged children are therefore the main target for preventive chemotherapy, consisting of a single 40 mg/kg oral dose of PZQ, as recommended by the World Health Organization (WHO) [5, 9, 10]. PZQ is the drug of choice because it is safe and efficacious against the adult stages of all *Schistosoma* species [11]. The efficacy of PZQ is typically expressed as a cure rate (CR) and often also as an intensity reduction rate (IRR), both based on pre- and post-treatment data. Reported CRs in school-aged children range between 42% and 79% for *S*. *mansoni* and between 37% and 93% for *S*. *haematobium* after a single 40 mg/kg oral dose of PZQ [12–14]. Following a closely spaced second dose of PZQ, considerably higher CRs are reported; 91% for *S*. *mansoni* [12, 15, 16] and 99% for *S*. *haematobium* [12].

Most studies reporting on the efficacy of PZQ have used microscopy-based methods, such as urine filtration for *S*. *haematobium* and the stool-based Kato-Katz (KK) technique for *S*. *japonicum* and *S*. *mansoni*. However, these methods lack sensitivity, especially for detection of low-intensity infections [17, 18]. Hence, reported CRs based on these parasitological methods are likely an overestimation [19, 20]. From a public health perspective, the absence or a significant reduction in the number of *Schistosoma* eggs is essential as they are causing morbidity and keep transmission ongoing. However, from an individual health care perspective, worm absence (cure) is more important. It is known that PZQ targets adult worms, therefore a direct determination of PZQ efficacy would be to measure the number of worms instead of eggs (which are usually used as a proxy for worm burden) with a highly accurate diagnostic tool. The field-applicable and commercially available point-of-care circulating cathodic antigen (POC-CCA) urine test, which identifies active worm infections by detection of schistosome CCA in urine, has shown a higher sensitivity for detecting *S*. *mansoni* infections than the KK technique [20–22]. It is now being recommended for surveillance and mapping of prevalence of intestinal schistosomiasis [10, 18, 21, 23].

In addition to the possible overestimation of CRs due to insensitive diagnostic tools, the limited activity of PZQ on immature worms as well as continuing reinfection might have led to an underestimation of the efficacy [24–27]. Furthermore, the short metabolic half-life of PZQ might also limit its effectivity [28]. In areas with ongoing transmission, where repeated infections and hence the presence of schistosomula in the human body is likely, repeating PZQ treatment a few weeks after the first dose might increase its overall effectiveness for parasitological cure [12, 29].

In the current study, we assessed the effect of multiple doses of PZQ on parasite clearance and tolerance in school-aged children from Côte d’Ivoire with a confirmed *S*. *mansoni* infection. As primary outcome we determined the difference in CR of a single versus four repeated doses of PZQ, measured by the KK technique in stool samples 10 weeks after the first treatment. Secondary outcomes included CR measured by the POC-CCA test, IRR by KK and POC-CCA, and safety of repeated PZQ treatments. Given the paucity of highly effective control measures for schistosomiasis, the results of our study are essential to assess the most optimal PZQ strategy from a public health control and best-care perspective.

## Methods

### Ethics statement

Ethics approval was obtained from the Comité National d’Éthique des Sciences de la Vie et de la Santé de Côte d’Ivoire (CNESVS; reference no. 091-18/MSHP/CNESVS-km, date of approval 27 June 2018), the Direction de la Pharmacie, du Médicament et des Laboratoires de Côte d’Ivoire (DPML; reference no. 99433/MSPH/DGS/DPML/DAR and clinical trial number ECCI00618, date of approval 22 October 2018), and the Ethics Committee of the Leiden University Medical Center in the Netherlands (P16.254, date of approval 11 January 2017). Communities and health authorities were informed on the purpose and procedures of the study. Participating children were informed about the objectives, procedures, and potential risks and benefits of the study using lay terms. Written informed consent was obtained from children’s parents or guardians, while children provided oral assent. The trial is registered at ClinicalTrials.gov (registration no. NCT02868385).

### Study design and participants

We conducted an open-label, randomized controlled trial with two arms in which children aged 5–17 years from three villages located in the Taabo health district in south-central Côte d’Ivoire [30] were included.

The trial was conducted from October 1, 2018 to January 14, 2019. In the first month of the study (October 2018), children were assessed for eligibility during a baseline screening. Children who were found positive for *S*. *mansoni* by KK and POC-CCA and egg-negative for *S*. *haematobium* by urine filtration were eligible. A detailed description of the inclusion and exclusion criteria is provided in the study protocol published elsewhere [31].

Eligible children were randomized into the ‘standard treatment’ group, receiving a single PZQ treatment at baseline, or the ‘intense treatment’ group, receiving PZQ treatment at baseline and again at two, four, and six weeks after the initial dose, totalling four treatments with two-week intervals in the intense treatment group. Due to logistic reasons and school holidays, final sample collection had to be postponed from 8 weeks (as described in the study protocol [31]) to 10 weeks after baseline treatment (see S1 Fig).

### Randomization and masking

Eligible school-aged children were randomly assigned to the standard or intense treatment group, as described elsewhere [31]. Participants as well as nurses and the study physician were not blinded to the treatment assignments, while laboratory technicians and investigators were blinded to the treatment assignments.

### Outcomes

The primary outcome was the difference in CR of a single versus four repeated PZQ treatments, based on KK in the intention-to-treat population. CR was defined as the proportion of children being *S*. *mansoni* egg-positive at baseline who became egg-negative 10 weeks after the first treatment. Secondary outcomes included the *S*. *mansoni* infection percentage positivity and intensity over time based on KK and POC-CCA, the CR based on POC-CCA, the IRR (defined as the percentage reduction in the median intensity, either expressed by eggs per gram of stool (EPG) or by visual score of the POC-CCA, of the positive individuals, 10 weeks after the first treatment, based on KK and POC-CCA, and safety of a single or multiple doses of PZQ).

### Procedures

Detailed descriptions of field and laboratory procedures are provided in the published study protocol [31]. In brief, during the baseline survey, single urine and single stool samples were collected from each participating child. Urine samples were subjected to POC-CCA (batch #170522062; Rapid Medical Diagnostics, Pretoria, South Africa) using the semi-quantitative scoring method called ‘G-scores’ [32]. With this POC-CCA batch, the provided quality control (QC) standard-series S0, S1, S2, and S3 resulted in a G1, G4, G8, and G10, respectively (see standard operating procedure (SOP), provided as an appendix in Casacuberta-Partal et al. [32]). To exclude the most abundant *S*. *haematobium* infections, urine filtration was performed on single baseline urine samples. Stool samples were processed using the KK technique with triplicate 41.7 mg thick smears prepared from each sample, as described previously [31, 33].

To assess treatment efficacy, additional urine and stool samples were collected from each participating child weekly and two-weekly, respectively, at eight time points over a period of 10 weeks. At each time point, all urine and stool samples were subjected to POC-CCA and KK, respectively, as described above.

### Treatment and monitoring of adverse events

At baseline, all included children were given PZQ (600 mg tablets; Biltricide, Bayer, Abidjan, Côte d’Ivoire), according to the calculated dose per kg of bodyweight (40 mg/kg, weight measured by a Seca 877 digital scale). Prior to treatment, breakfast was provided to each child. After sample collection, directly observed treatment was applied by the study physician and was accompanied with water and lunch, provided by the research team. Children allocated to the intense treatment group were re-treated with PZQ at 2, 4, and 6 weeks after the first treatment.

After treatment, children remained under medical supervision for at least 3 hours and adverse events were recorded. If needed, symptomatic treatment for adverse events was provided by the study physician. In case vomiting occurred within 1.5 hours, children were re-administered a dose of PZQ. Twenty-four hours post-treatment, children were interviewed about the occurrence of adverse events. An adverse event was defined as any undesirable sign, symptom, or disease occurring to a participant during the study, whether or not related to PZQ treatment. Intensity of adverse events was graded by the study physician as mild, moderate, or severe, following guidelines by the European Medicine Agency.

### Statistical analysis

A detailed description of the sample size calculation is given elsewhere [31]. In brief, to detect an increase in CR from 66% after a single PZQ treatment [12] to 99% after four repeated PZQ treatments with a two-sided 5% significance level and a power of 90%, a minimum sample size of 30 children per group was required [31, 34]. To account for follow-up losses, expected due to the intense weekly follow-up, the sample size was increased to 100 children in each group, hence 200 children in total. Assuming a *S*. *mansoni* infection prevalence of approximately 25% based on KK (Taabo health and demographic surveillance site survey carried out in February 2016), at least 1,000 children needed to be screened in order to obtain a minimum of 200 KK-positive children in the Taabo region.

Data were double entered by two well-trained data entry clerks and managed using a REDCap electronic data capture tool hosted at Leiden University Medical Center (Leiden, the Netherlands), via Emory University (Atlanta, United States of America) [35, 36]. Descriptive statistics were performed using IBM Statistical Package for Social Sciences version 25 (SPSS Inc., Chicago, United States of America).

Infection intensity, as expressed by EPG, was calculated by multiplying the sum of egg counts from triplicate KK thick smears by a factor of 8. Intensity of infection was classified according to WHO guidelines into light (1–99 EPG), moderate (100–399 EPG), and heavy (≥400 EPG) [5]. POC-CCA G-scores were classified into negative (G1), trace (G2-3, conservatively considered as negative in the analysis presented here), 1+ (G4-5), 2+ (G6-7), or 3+ (G8-10) [10, 22, 32, 37].

To determine the prevalence over time as well as CRs (based on KK and POC-CCA) and IRRs (based on KK), mixed effects models were employed to take into account the correlation between the different measurements from the same individual [38–40]. For the prevalence, we used mixed effects logistic regression where prevalence was modeled as a function of time, treatment group and their interaction. In the case of KK, the time variable was taken as categorical, while for POC-CCA we modeled progression over time using natural cubic splines with four knots. For the KK-based IRR, we used a zero-inflated negative binomial mixed model where the logarithm of the mean egg counts is modeled as a function of time (using natural cubic splines), treatment group and their interaction. For POC-CCA, the IRRs could not be obtained from the mixed effects model, as the output (G-score) is not a continuous variable. Therefore, the IRR based on POC-CCA was calculated according to WHO guidelines (1 − [arithmetic mean after treatment/arithmetic mean before treatment]) × 100 [41]. In all models, the within subject correlation was modeled using a random intercepts term. The models used provide results under the missing at random assumption for the missing data which is valid in this study. All analyses were done in R (version 3.43) using the GLMMadaptive package. CR and IRR estimated from the model are given with their corresponding 95% pointwise confidence intervals (CIs). *P*-values <0.05 were considered to indicate statistical significance.

## Results

**Fig 1:**
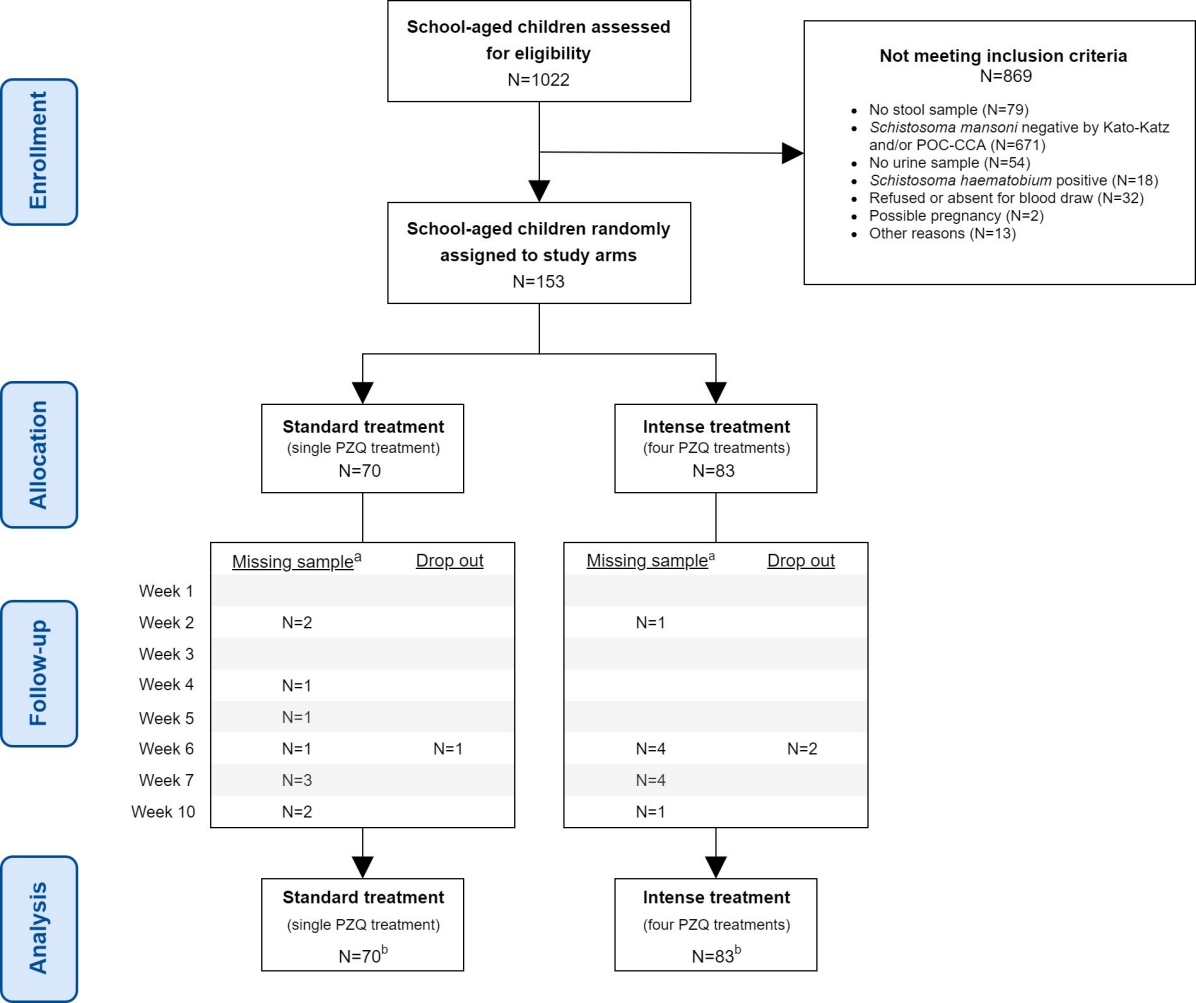
Trial profile. a. Sample (urine and/or stool) not provided. b. Intention-to-treat analysis.

Fig 1 shows the study flow. At baseline, 1,022 children aged 5–17 years were assessed for eligibility. Of these, 153 had a detectable *S*. *mansoni* infection and met the inclusion criteria. They were randomly assigned to one of the two study arms; 70 were assigned to the standard treatment group, and 83 were assigned to the intense treatment group. Regular randomization was performed instead of block randomization, and hence, the size of the two groups differed. At baseline, all 153 children (100%) received treatment. Three children (one in the standard treatment group and two in the intense treatment group) were lost to follow-up from week 6 onwards because they moved out of the study region during the follow-up period (see S1 Table). In the intense treatment group, compliance to each following treatment was unexpectedly high, from 100% in week 2 (second treatment) to 98% in week 6 (fourth and final treatment). All 153 children were included in the intention-to-treat analysis.

The demographic and parasitological baseline data for the participating children are summarized in Table 1. The median age and sex of children were balanced among the two groups. In both groups, most of the children had a light to moderate *S*. *mansoni* infection, while heavy infection intensities were observed in 18 (26%) children in the standard treatment group compared to 12 (14%) children in the intense treatment group. At pre-treatment, the median fecal egg count was 172 EPG and the median G-score in urine was 6 in the standard treatment group, and 128 EPG and G-score 7 in the intense treatment group, respectively.

**Table 1:** Baseline characteristics of the standard treatment group and the intense treatment group in a randomized trial. The trial was conducted in late 2018 among school-aged children in south-central Côte d’Ivoire and compared single versus four repeated PZQ treatments against *S*. *mansoni*.

	Standard treatment group (1x PZQ)	Intense treatment group (4x PZQ)
	N = 70	N = 83
Age, years	10.5 (9–12)	10.0 (9–12)
Weight, kg	32.2 (27.3–38.4)	32.0 (26.5–38.0)
Height, cm	137 (130–145)	140 (128–146)
Hemoglobin (g/dl)	11.3 (10.8–11.8)	11.3 (10.8–12.0)
Sex		
Boys	43 (61.4%)	51 (61.4%)
Girls	27 (38.6%)	32 (38.6%)
Village		
Ahouaty	35 (50.0%)	41 (49.4%)
N’Denou	27 (38.6%)	33 (39.8%)
Singrobo	8 (11.4%)	9 (10.8%)
Infection intensity		
Kato-Katz		
Light (1–99 EPG)	24 (34.3%)	35 (42.2%)
Moderate (100–399 EPG)	28 (40.0%)	36 (43.4%)
Heavy (≥400 EPG)	18 (25.7%)	12 (14.4%)
POC-CCA^a^		
1+	16 (22.9%)	22 (26.5%)
2+	38 (54.2%)	50 (60.2%)
3+	16 (22.9%)	11 (13.3%)

Data are median (IQR) or n (%). Abbreviations: EPG, eggs per gram of stool; IQR, interquartile range; POC-CCA, point-of-care circulating cathodic antigen.

^a^ POC-CCA positive G-scores were classified into 1+ (G4-5), 2+ (G6-7) or 3+ (G8-10).

### Prevalence over time

**Fig 2:**
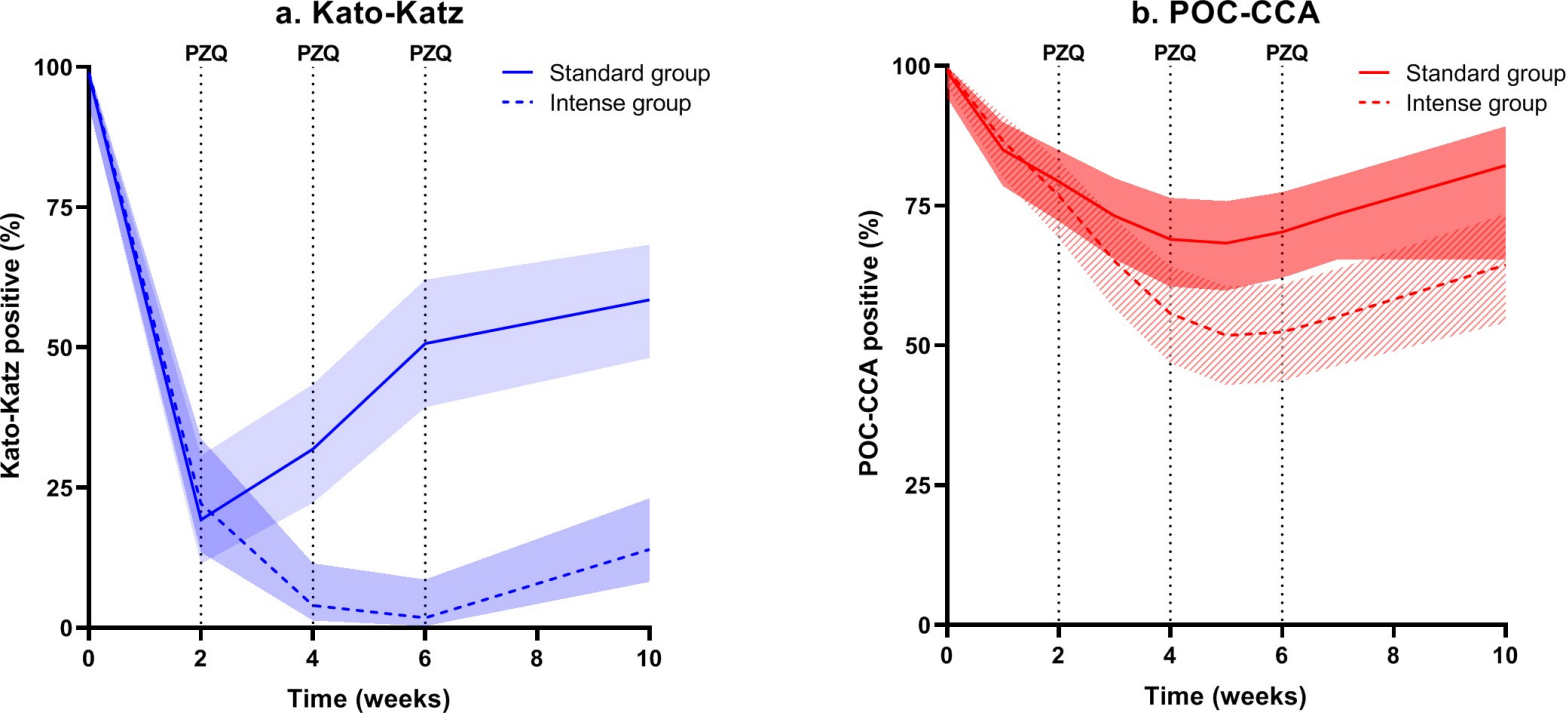
Prevalence over time (with corresponding pointwise 95% confidence intervals) estimated from the mixed effects logistic regression model. Data pertain to (a) triplicate Kato-Katz (KK) thick smears from a single stool sample and (b) single point-of-care circulating cathodic antigen (POC-CCA) urine test in the standard treatment group (single dose of PZQ, solid line) and the intense treatment group (four doses of PZQ at W0, W2, W4, and W6, dashed line).

Fig 2 illustrates the percentage of *S*. *mansoni* positives over time based on KK (Fig 2A) and on POC-CCA (Fig 2B). In the standard treatment group, the overall *S*. *mansoni* prevalence based on triplicate KK thick smears was decreased from 100% to 58% (95% CI 48–68%), measured 10 weeks after treatment. In the intense treatment group, who received a total of four doses of PZQ, the prevalence decreased to 14% (95% CI 8–23%), measured 10 weeks after the first treatment. Based on POC-CCA, measured at the final follow-up time point, 82% (95% CI 72–89%) and 64% (95% CI 54–74%) POC-CCA positives were observed in the standard and intense treatment group, respectively.

### Intensity of infection over time

**Fig 3:**
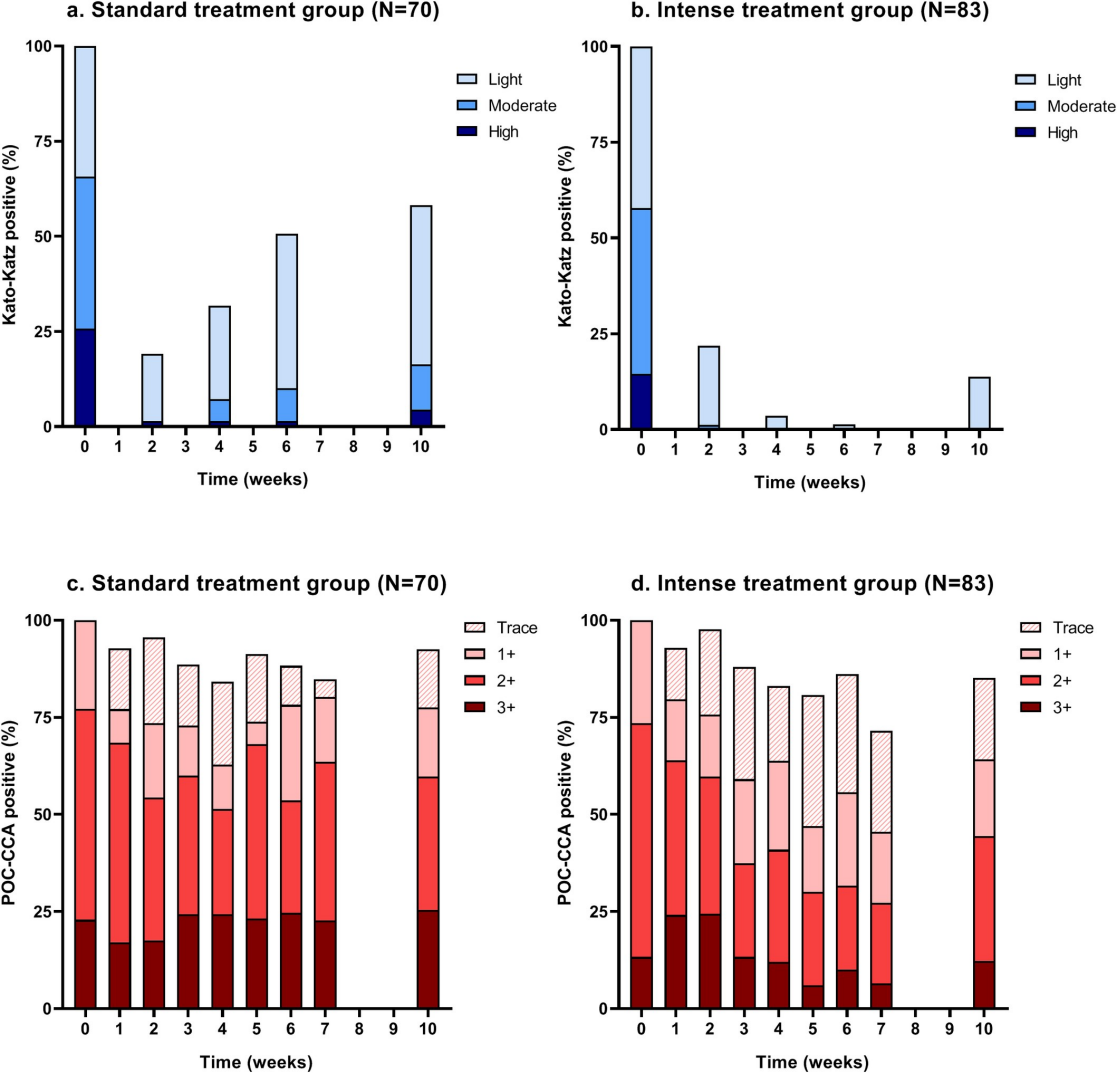
Intensity of infection. Data are based on triplicate Kato-Katz (KK) thick smears from a single stool sample (a, b) and single point-of-care circulating cathodic antigen (POC-CCA) urine test (c, d) in the standard treatment group (single dose of PZQ) and the intense treatment group (four doses of PZQ at W0, W2, W4, and W6).

The intensity of infection over time based on KK and POC-CCA is shown in Fig 3 (see also S2 Fig and S3 Fig). Based on the KK technique (Fig 3A and 3B), most of the remaining infections after the first treatment were of low intensity. In the standard treatment group, the proportion of low, moderate and, to a smaller extent, heavy intensity infections showed an increase 10 weeks after treatment. In the intense treatment group, a small proportion of infections of low intensity were observed at the final time point. Based on POC-CCA (Fig 3C and 3D), the overall prevalence did not change dramatically and the proportion of 3+ scores (indicating high infection level) remained similar over time in the standard treatment group as well as in the intense treatment group. The proportion of POC-CCA negatives (including traces) increased over time, particularly in the intense treatment group. In both groups, most children remained POC-CCA positive at the final time point. A positive correlation was observed between fecal egg counts and POC-CCA visual scores before treatment (Spearman’s rho = 0.44, *P*<0.01) (S4 Fig).

### Cure rate

In the standard treatment group, CR based on triplicate KK thick smears was 42% (95% CI 31–53%) (Table 2; see also S2 Table). A significantly higher CR (86%, 95% CI 75–92%) was observed in the intense treatment group, (*P*<0.01; primary outcome, both CRs measured 10 weeks after first treatment). When using the same time interval post-treatment to compare CRs between the two groups, i.e., four weeks after the first treatment for the standard treatment group and four weeks after the fourth treatment for the intense treatment group, the observed CR in the standard treatment group was 68% (95% 57–78%) compared to 86% (95% CI 75–92%) in the intense treatment group (*P*<0.01).

**Table 2:** Cure rate (CR) and intensity reduction rate (IRR) of a single (standard treatment group) and four (intense treatment group) repeated PZQ treatments in school-aged children infected with *S*. *mansoni*. Data are based on triplicate Kato-Katz (KK) thick smears from a single stool sample and single point-of-care circulating cathodic antigen (POC-CCA) urine test.

	Standard treatment group (1x PZQ) N = 70	Intense treatment group (4x PZQ) N = 83
**Kato-Katz**		
Infected children before treatment	70	83
Cured children 10 weeks after first treatment	28	69
CR^a^^,^^b^	41.6% (95% CI 31.1–52.9%)	86.0% (95% CI 75.4–92.4%)
Cured children 4 weeks post-treatment^c^	47	69
CR^b^	68.2% (95% CI 57.1–77.6%)	86.0% (95% CI 75.4–92.4%)
Median EPG^d^		
Before treatment	172	128
10 weeks after first treatment	64	8
4 weeks post-treatment^c^	36	8
Arithmetic mean EPG		
Before treatment	298.2	242.7
10 weeks after first treatment	97.7	3.2
IRR^e^	72.3% (95% CI 54.6–83.1%)	95.1% (95% CI 85.1–98.4%)
4 weeks post-treatment^c^	45.8	3.2
IRR^e^	83.3% (95% CI 68.9–91.2%)	95.1% (95% CI 85.1–98.4%)
**POC-CCA**		
Infected children before treatment	70	83
Cured children 10 weeks after first treatment	15	29
CR^b^	17.9% (95% CI 11.3–27.2%)	35.7% (95% CI 26.4–46.1%)
Cured children 4 weeks post-treatment^c^	26	29
CR^b^	31.2% (95% CI 23.4–40.2%)	35.7% (95% CI 26.4–46.1%)
Median G-score^d^		
Before treatment	6	7
10 weeks after first treatment	7	6
4 weeks post-treatment^c^	6	6
Arithmetic mean G-score		
Before treatment	6.4	6.3
10 weeks after baseline treatment	5.8	4.6
IRR^f^	9.3%	27.0%
4 weeks post-treatment^c^	5.1	4.6
IRR^f^	20.3%	27.0%

Abbreviations: CR, cure rate; EPG. eggs per gram of stool; IRR, intensity reduction rate; POC-CCA, point-of-care circulating cathodic antigen

^a^ Primary outcome

^b^ CR as calculated from the model

^c^ Measured four weeks after first treatment for the standard treatment group, and four weeks after the fourth treatment for the intense treatment group

^d^ Median of the positives

^e^ IRR based on the reduction in mean EPG as calculated from the model

^f^ IRR based on the reduction in mean POC-CCA G-score as calculated manually

POC-CCA-based CRs were much lower compared to CRs based on the KK technique; only 18% (95% CI 11–27%) in the standard treatment group and 36% (95% CI 26–46%) in the intense treatment group (*P*<0.01). Using the 4-week post-treatment time points, CRs were similar in both groups; 31% (95% CI 23–40%) in the standard treatment group and 36% (95% CI 26–46%) in the intense treatment group (*P* = 0.23).

### Intensity reduction rate

Based on the KK technique, the IRR in the standard treatment group was 72% (95% CI 55–83%), compared to 95% (95% CI 85–98%) in the intense treatment group (*P*<0.01). When using the same time interval post-treatment to compare IRRs between the two groups (4 weeks), the observed IRR in the standard treatment group was 83% (95% CI 69–91%) versus 95% (95% CI 85–98%) in the intense treatment group (*P*<0.01). The decrease in the mean POC-CCA G-score was larger in the intense treatment group compared to the standard treatment group, resulting in an IRR of 27% and 9%, respectively (measured 10 weeks after first treatment). When using the same time interval post-treatment for the standard treatment group, the IRR was 20%.

### Safety of PZQ

Observed and reported (within 3 hours) adverse events are summarized in Table 3, stratified by treatment group and follow-up time point. After the first treatment, stomach ache was the most common adverse event (overall 38%), followed by headache (overall 5%) and vomiting (overall 3%). Most of the adverse events were mild and all of them resolved 24 hours after treatment. Adverse events decreased with subsequent treatments in the intense treatment group.

**Table 3:** Main type of adverse events observed and reported 3 hours after PZQ administration in *S*. *mansoni*-infected children in the standard treatment group and the intense treatment group.

	Standard treatment group		Intense treatment group							
	First treatment W0		First treatment W0		Second treatment W2		Third treatment W4		Fourth treatment W6	
	N = 70		N = 83		N = 82		N = 82		N = 78	
**Adverse events**										
Stomach ache	25	36%	33	40%	25	30%	22	27%	10	13%
Headache	3	4%	5	6%	14	17%	4	5%	2	3%
Vomiting	3	4%	2	2%	2	2%	4	5%	2	3%
Dizziness	2	3%	2	2%	3	4%	9	11%	6	8%
Diarrhea	2	3%	1	1%	0		0		0	
Nausea	0		0		0		1	1%	1	1%

## Discussion

Based on stool microscopy, we observed a significantly higher CR after four closely spaced PZQ treatments compared to a single dose, measured 10 weeks after the first treatment, without any difference in the frequency and severity of adverse events. Employing the POC-CCA test, the observed CRs were considerably lower compared to KK, even after four repeated treatments, indicating that worms are still present and that PZQ might be less efficacious than previously published.

Our aim of administering PZQ four times at 2-week intervals was to achieve a high CR, as this approach not only targets adult schistosomes, but also the immature forms, which were not yet drug-susceptible during the first treatment [12, 29, 42]. Indeed, 10 weeks after the baseline survey, four repeated treatments resulted in a significantly higher CR than a single treatment based on the KK technique, but failed to cure all infections. The primary outcome according to the study protocol, i.e., the difference in CR of a single versus four repeated PZQ treatments, was calculated by comparing the prevalence of infection at baseline and 10 weeks after the first treatment [31]. This implied that the time interval after treatment was not the same for both groups; 10 weeks after the single treatment in the standard treatment group (allowing for a 10-week period of possible worm maturation, worm recovery, as well as renewed parasite exposure and re-infection) versus four weeks after the fourth treatment in the intense treatment group. To render the comparison between the groups more representative, CRs were also determined using the same time interval for both groups, i.e., for the standard treatment group taking four weeks after the first treatment as the final time point. While the CR in the standard treatment group was significantly higher four weeks after treatment compared to the CR obtained 10 weeks after treatment, there was no significant difference in the CRs between the standard treatment group and the intense treatment group four weeks after the last treatment, measured with both KK and POC-CCA. Hence, following this evaluation approach, there is no indication that four repeated PZQ treatments outperform a single treatment in curing schistosomiasis.

Moreover, although four repeated PZQ treatments resulted in a statistically significantly higher CR when utilizing the KK technique compared to POC-CCA, the estimated CR was considerably lower than what we had expected [31], and the proportion of KK-positives increased again from 1% before the fourth treatment to 14% four weeks after the fourth treatment. This might indicate continued parasite exposure, ongoing re-infection, and worm maturation after treatment over the course of the study, not excluding possible PZQ resistance.

Compliance to treatment was very high at each treatment, most likely due to the relatively mild and short-lived adverse events in combination with the commitment of the field and laboratory team, and enthusiastic participation of children. At each treatment time point, directly observed treatment was applied by the study physician. We therefore conclude that the observed increase in proportion of KK-positives at the final time point in the intense treatment group cannot be explained by the fact that some children might not have taken the (repeatedly) administered drugs, but points to parasite survival in the host or rapid reinfection.

To more accurately assess the CR, the POC-CCA test was employed in addition to KK. CRs based on POC-CCA were significantly lower in both groups compared to CRs based on the KK technique. Even though the number of POC-CCA negatives increased after four treatments, still more than half of the participants remained positive (traces conservatively considered as negative), indicating that active *Schistosoma* infections were still present in our cohort or new infections occurred within short periods.

Previous studies have demonstrated false positive POC-CCA results in people with urinary tract infections [43] as well as potentially in pregnant women [44] and new born babies. In our study, no urinary tract infections were noted, two female participants were excluded because of possible pregnancy, and only children aged 5 years and above were included. To further minimize the inclusion of false positives, traces were considered as negative in our analysis. Recent studies have shown that prevalence estimates of *S*. *mansoni* below 20% according to the KK technique might correspond to POC-CCA prevalence estimates that are 3- to 4-fold higher [18, 45]. Furthermore, studies applying latent class analysis to determine the performance of diagnostic assays have shown that the POC-CCA test has a considerably greater sensitivity and a comparable specificity than the KK technique, especially when traces are considered negative [20, 46]. In contrast, other studies have shown that POC-CCA might be of limited use to diagnose *S*. *mansoni* infection, especially in low endemic areas [47]. More accurate diagnostic methods, such as PCR [48, 49] or the upconverting phosphor lateral flow (UCP-LF) assay detecting circulating anodic antigen [50], both being well established laboratory-based assays, could be used to determine prevalence more accurately.

It is important to note that the two diagnostic methods employed in our study detect different *Schistosoma* life-cycle stages, namely eggs in case of KK thick smears examined under a microscope and antigens derived from adult worms in case of the POC-CCA urine test. Finding a high proportion of individuals positive by POC-CCA after treatment, while no eggs are detected by KK, indicates that the infection is not fully cured. Mature worms could have been affected by PZQ but not killed, resulting in the (temporary) reduction or cessation of fecal egg excretion [13], while still excreting CCA detectable by POC-CCA. Furthermore, individuals can harbor living (single sex) worms with only sporadically excreting eggs in stool or with no detectable eggs at all [51–54]. CCA might also originate from new infections or immature worms [55] or perhaps from dead worms that were killed by PZQ. Lastly, PZQ treatment could have resulted in a reduction of fecundity, indicating that egg-based diagnostic methods will overestimate the reduction in worm burden [56, 57]. However, even with the presence of low worm numbers, which excrete relatively few eggs, there is a continued risk of pathology [58, 59], as eggs could retain in the host tissue, where they induce inflammatory responses resulting in ‘subtle morbidity’ [60].

Little is known about the transmission of schistosomes in the study setting of south-central Côte d’Ivoire; transmission is suspected to be ongoing, given the number of observed cases and the frequent surface water contact of the inhabitants with the man-made Lake Taabo [27, 30]. This trial focused on simply measuring the efficacy of PZQ and did not include sanitation and behavioral interventions, which in this specific setting would have had an additional impact on the number and intensity of *Schistosoma* infections. Moreover, since this study focused on a subset of school-aged children, other children in the same age range, as well as preschool-aged children or adolescents/adults from the community would still serve as a reservoir of infection and therefore contribute to ongoing transmission.

The CR and IRR as determined by the KK technique were higher after four treatments compared to a single treatment, both at four and 10 weeks after treatment. This observation suggests that repeated treatment has an added value on reducing the number of infections and *Schistosoma*-related infection intensity and thus morbidity in areas where people are likely infected with different developmental stages of the parasite and rapid re-infection is obvious. However, since microscopy lacks sensitivity, especially in infections of low intensity and post-treatment settings, not being able to detect eggs does not necessarily mean that the infection is cured and that all worms have been killed. In contrast to the high IRR based on the KK technique, only a minor reduction in POC-CCA-based infection intensity was observed, which did not increase significantly after four repeated PZQ treatments. This contradicts previous studies that indicated a decrease in POC-CCA intensity score rapidly after treatment [20, 47, 61]. More accurate diagnostic methods, such as the UCP-LF CAA test or quantitative PCR could be applied to more accurately determine the reduction in intensity.

Previous studies indicate that the frequency and severity of adverse events is related to *Schistosoma* infection intensity, with more events reported in infections with a heavy intensity [62, 63]. Over the course of the trial, mostly mild and short-lived adverse events were observed, which presumably can be attributed to the relatively low intensity of infection in our study population. Overall, repeated PZQ treatment was well tolerated, indicating that repeated PZQ treatment can be considered as safe. Repeated PZQ treatment might help to enhance the control of schistosomiasis. This should not preclude the notion that treatment, whether single or repeated, should always be combined with other control measures, such as behavior change, sanitation, safe water, and snail control interventions in order to bolster the effect of PZQ and to move toward interruption of transmission.

## Conclusion

Based on stool microscopy using the KK technique, four repeated doses of 40 mg/kg PZQ at 2-week intervals resulted in a CR against *S*. *mansoni* infection of 86%, as determined 10 weeks after the initial treatment. When using the more sensitive POC-CCA test, the observed CR was significantly lower (27%), indicating that PZQ might not be as efficacious as previously reported. The same trend is shown when efficacy is expressed as IRR, which highlights the relevance of accurate diagnostic methods in monitoring treatment efficacy as well as other control approaches (e.g. vaccine development).

This study signifies that the development and field implementation of reliable and more accurate diagnostic tools are essential to systematically map transmission intensity and measure efficacy of control strategies, ultimately providing rational guidance on the path toward elimination of schistosomiasis.

## Supporting information

S1 Checklist CONSORT Checklist. (DOC)

S1 Fig Schematic representation of follow-up, treatment and sampling procedures in the standard and intense treatment group. Adapted from the published study protocol.
(TIF)

S2 Fig **Intensity of infection of KK-positives over time based on triplicate thick smears from a single stool sample in the standard treatment group (single PZQ treatment) (a) and the intense treatment group (four repeated PZQ treatments at W0, W2, W4, and W6) (b)**.
(TIF)

S3 Fig **Individual intensity of infection over time based on triplicate Kato-Katz (KK) thick smears from a single stool sample (a, b) and single point-of-care circulating cathodic antigen (POC-CCA) urine test using visual scores (c, d) or G-scores (e, f) in the standard treatment group (single PZQ treatment) and the intense treatment group (four repeated PZQ treatments at W0, W2, W4, and W6)**.
(TIF)

S4 Fig Correlation between EPG (based on KK) and G-scores (based on POC-CCA) before treatment. (TIF)

S1 Table Detailed overview of missing samples and losses to follow-up in the standard treatment group and the intense treatment group. NA, not applicable. ^a^ Lost to follow-up.
(DOCX)

S2 Table Cure rate (CR) and intensity reduction rate (IRR) after one, two, three and four treatments with PZQ at two-week intervals in school-aged children infected with *S*. *mansoni* based on triplicate Kato-Katz (KK) thick smears from a single stool sample and single point-of-care circulating cathodic antigen (POC-CCA) urine test. Abbreviations: CR, cure rate; EPG, eggs per gram of stool; IRR, intensity reduction rate; NP, not possible; POC-CCA, point-of-care circulating cathodic antigen. ^a^ Number of infected children at baseline. ^b^ Measured 2 weeks post-treatment for one, two and three treatments and measured 4 weeks post-treatment for four treatments. ^c^ CR as calculated from the model based on the probability of being cured. ^d^ Median of the positives. ^e^ IRR based on the reduction in mean EPG as calculated from the model. ^f^ IRR based on the reduction in mean POC-CCA G-score as calculated manually.
(DOCX)
