# Supplementary figures and images for: Efficacy of single versus four repeated doses of praziquantel against Schistosoma mansoni infection in school-aged children from Côte d'Ivoire based on Kato-Katz and POC-CCA: An open-label, randomised controlled trial (RePST)
Source: PLoS Negl Trop Dis. 2020 Mar 20;14(3):e0008189. doi: 10.1371/journal.pntd.0008189 (PMC7112237; doi:10.1371/journal.pntd.0008189)

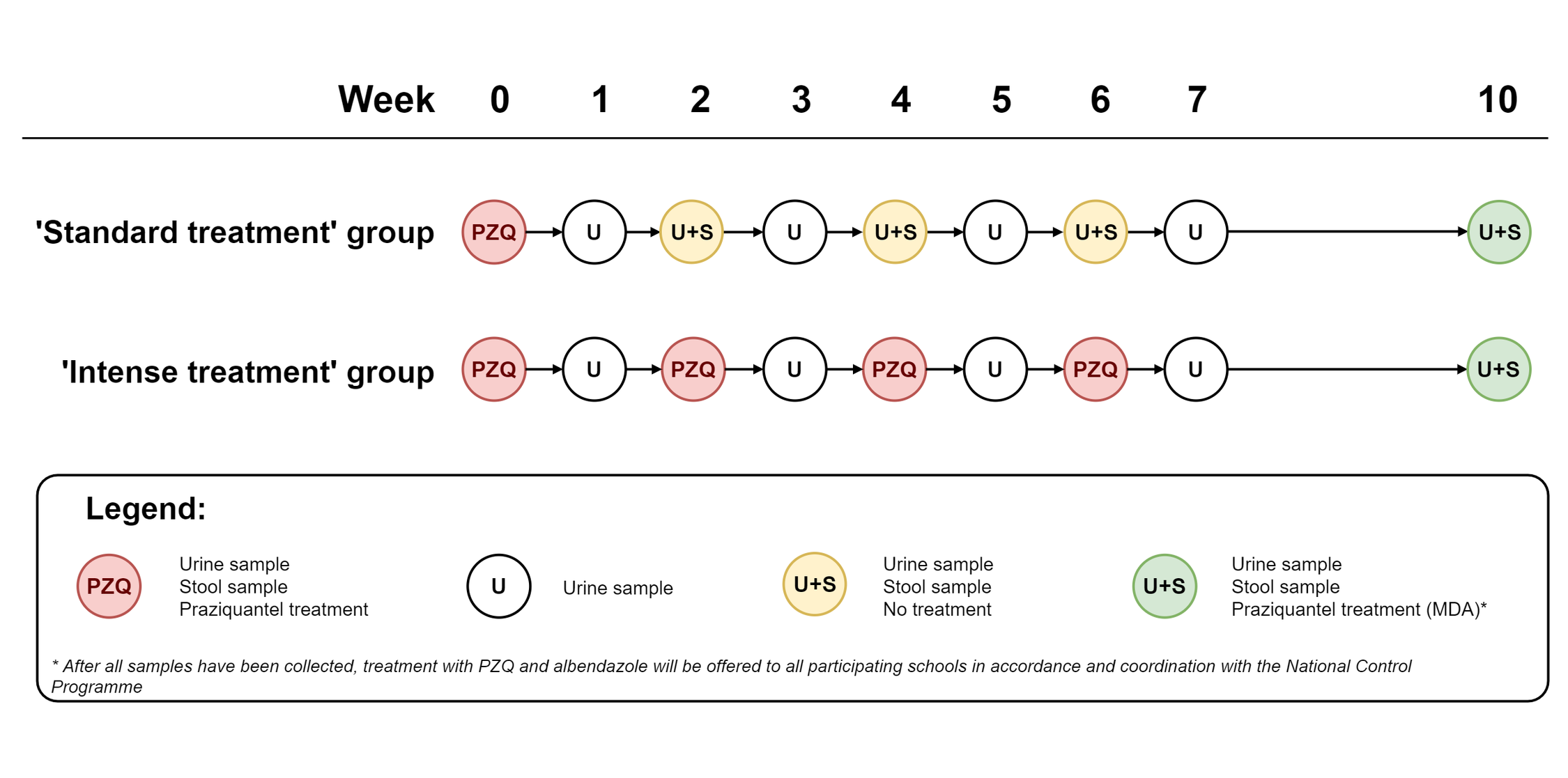

Supplement: S1 Fig — Adapted from the published study protocol. (TIF) [file pntd.0008189.s002.tif]

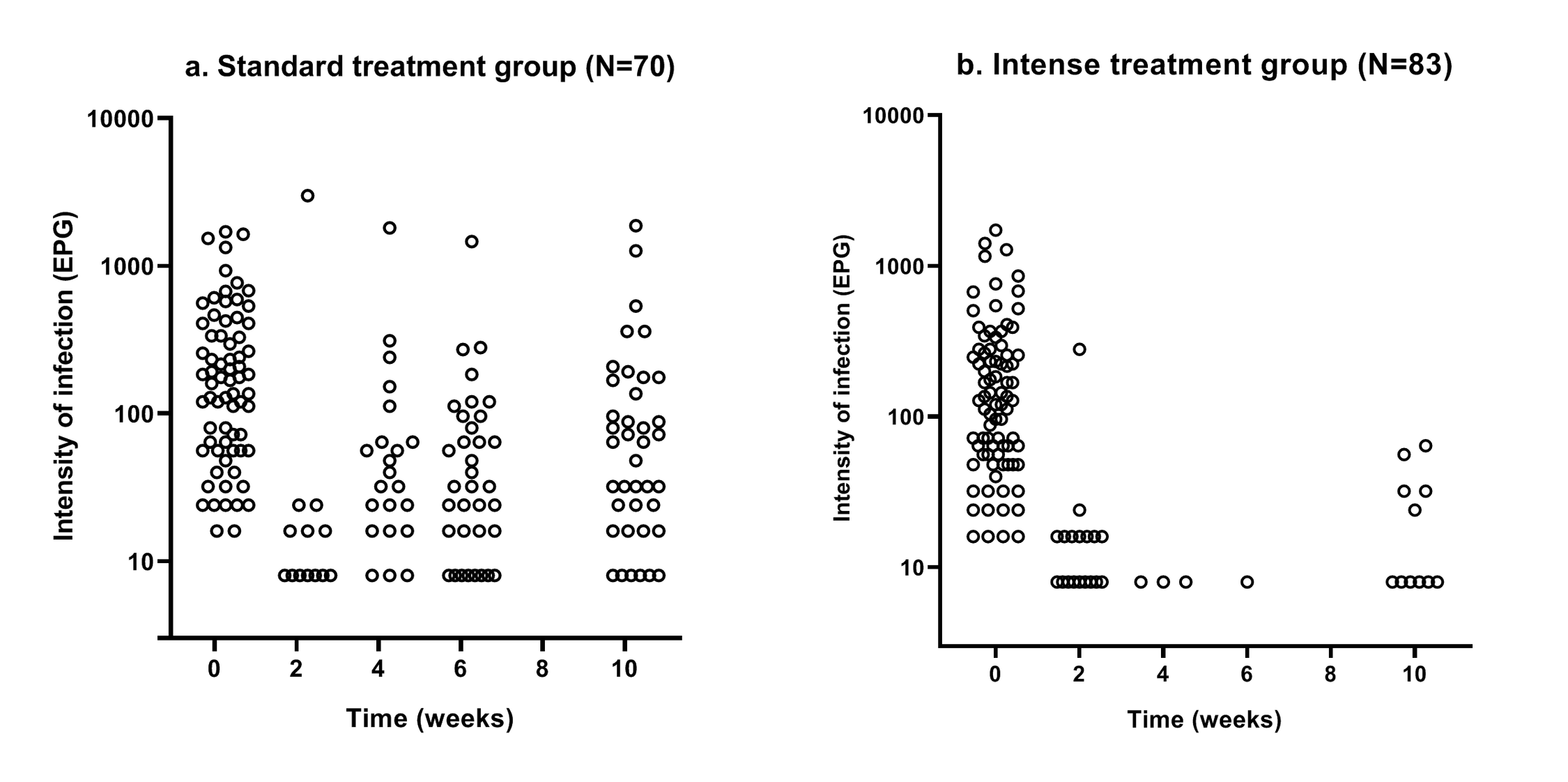

Supplement: S2 Fig — Intensity of infection of KK-positives over time based on triplicate thick smears from a single stool sample in the standard treatment group (single PZQ treatment) (a) and the intense treatment group (four repeated PZQ treatments at W0, W2, W4, and W6) (b). (TIF) [file pntd.0008189.s003.tif]

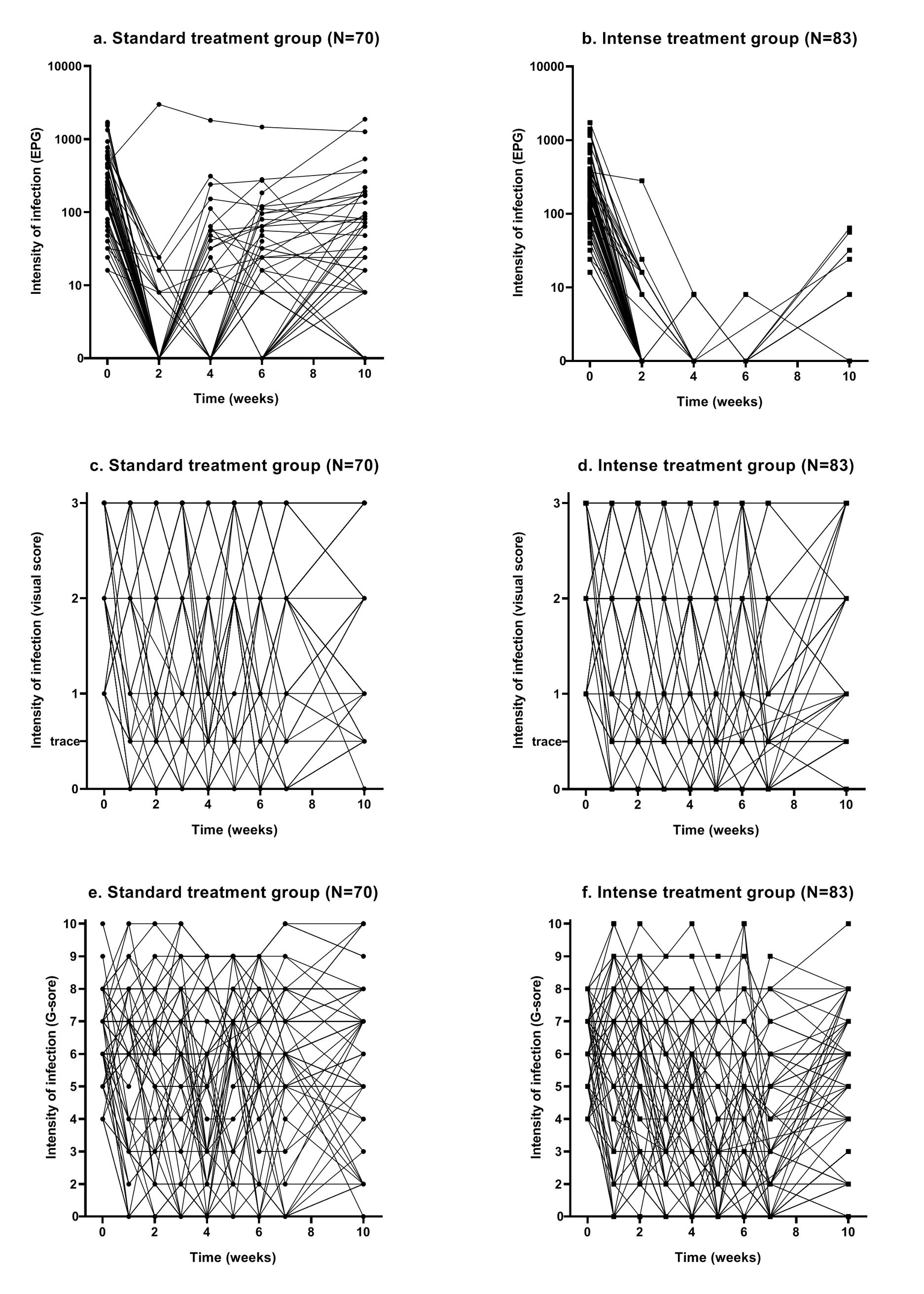

Supplement: S3 Fig — Individual intensity of infection over time based on triplicate Kato-Katz (KK) thick smears from a single stool sample (a, b) and single point-of-care circulating cathodic antigen (POC-CCA) urine test using visual scores (c, d) or G-scores (e, f) in the standard treatment group (single PZQ treatment) and the intense treatment group (four repeated PZQ treatments at W0, W2, W4, and W6). (TIF) [file pntd.0008189.s004.tif]

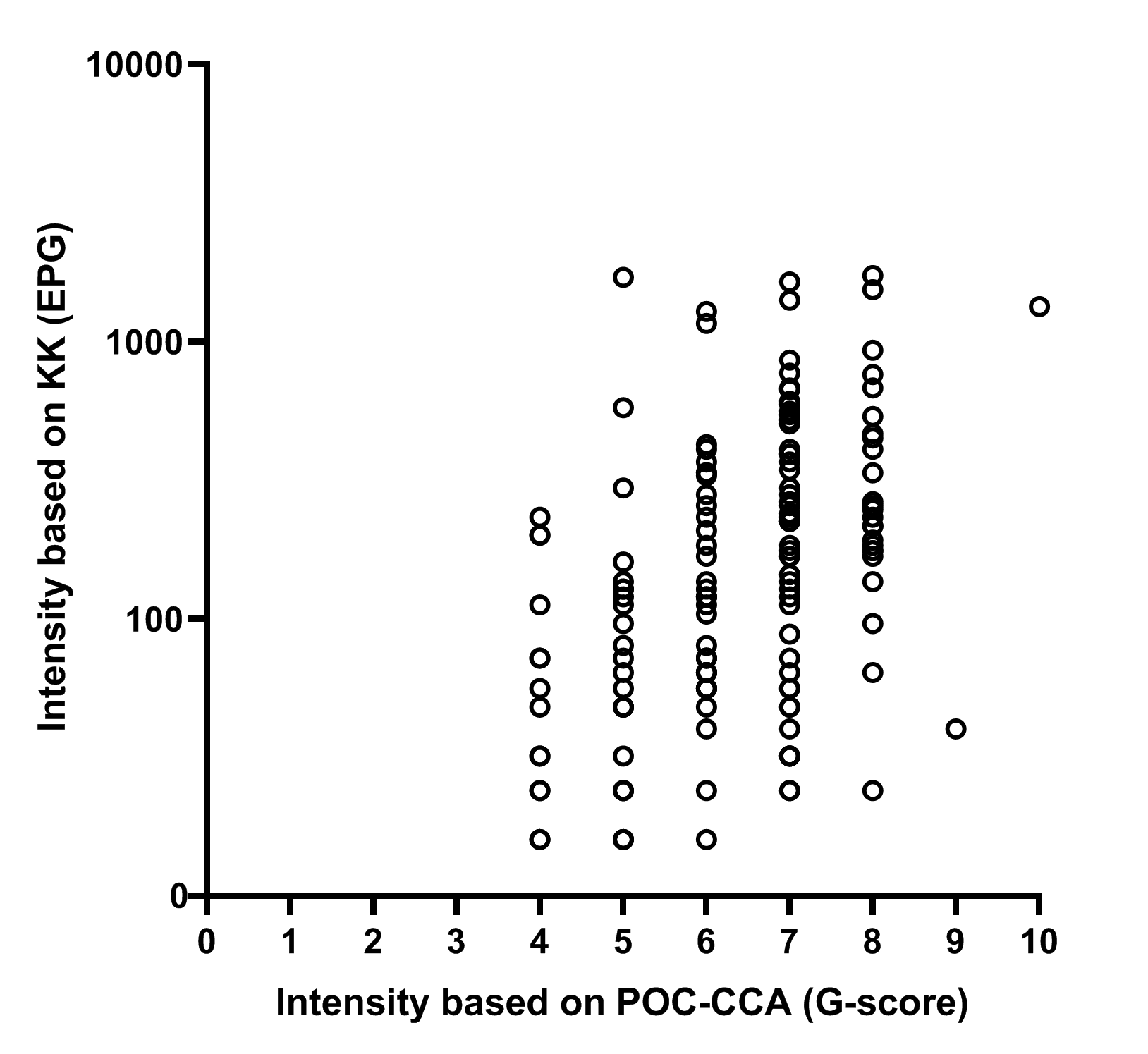

Supplement: S4 Fig — (TIF) [file pntd.0008189.s005.tif]
