# Supplementary material for: Efficacy of single versus four repeated doses of praziquantel against Schistosoma mansoni infection in school-aged children from Côte d'Ivoire based on Kato-Katz and POC-CCA: An open-label, randomised controlled trial (RePST)
Source: PLoS Negl Trop Dis. 2020 Mar 20;14(3):e0008189. doi: 10.1371/journal.pntd.0008189 (PMC7112237; doi:10.1371/journal.pntd.0008189)
Supplement: S1 Table — NA, not applicable. a Lost to follow-up. (DOCX) [file pntd.0008189.s006.docx]

**S1 Table. Detailed overview of missing samples and drop-outs during follow-up in the standard treatment group and the intense treatment group.**

|  | **Standard treatment group**  (1x PZQ)  N=70 | | | **Intense treatment group**  (4x PZQ)  N=83 | | |
| --- | --- | --- | --- | --- | --- | --- |
|  | Total | Missing | Participant ID | Total | Missing | Participant ID |
| **W0** |  |  |  |  |  |  |
| Received PZQ treatment | N=70 | N=0 |  | N=83 | N=0 |  |
| Provided stool sample | N=70 | N=0 |  | N=83 | N=0 |  |
| Provided urine sample | N=70 | N=0 |  | N=83 | N=0 |  |
| **W1** |  |  |  |  |  |  |
| Provided urine sample | N=70 | N=0 |  | N=83 | N=0 |  |
| **W2** |  |  |  |  |  |  |
| Received PZQ treatment | NA | NA |  | N=82 | N=1 | 2023 |
| Provided stool sample | N=68 | N=2 | 1155/2453 | N=82 | N=1 | 2023 |
| Provided urine sample | N=68 | N=2 | 1155/2453 | N=82 | N=1 |  |
| **W3** |  |  |  |  |  |  |
| Provided urine sample | N=70 | N=0 |  | N=83 | N=0 |  |
| **W4** |  |  |  |  |  |  |
| Received PZQ treatment | NA | NA |  | N=82 | N=1 | 2457 |
| Provided stool sample | N=69 | N=1 | 1105 | N=83 | N=0 |  |
| Provided urine sample | N=70 | N=0 |  | N=83 | N=0 |  |
| **W5** |  |  |  |  |  |  |
| Provided urine sample | N=69 | N=1 | 1535 | N=83 | N=0 |  |
| **W6** |  |  |  |  |  |  |
| Received PZQ treatment | NA | NA |  | N=78 | N=5 | 2050^a^/2149/2260/2282/2457^a^ |
| Provided stool sample | N=68 | N=2 | 1105^a^/2449 | N=77 | N=6 | 1425/2050^a^/2149/2282/2371/2457^a^ |
| Provided urine sample | N=69 | N=1 | 1105^a^ | N=79 | N=4 | 2050^a^/2149/2282/2457^a^ |
| **W7** |  |  |  |  |  |  |
| Provided urine sample | N=66 | N=4 | 1105^a^/1416/1549/2336 | N=77 | N=6 | 1243/2050^a^/2146/2149/2282/2457^a^ |
| **W10** |  |  |  |  |  |  |
| Provided stool sample | N=67 | N=3 | 1034/1105^a^/1155 | N=80 | N=3 | 1512/2050^a^/2457^a^ |
| Provided urine sample | N=67 | N=3 | 1034/1105^a^/1155 | N=81 | N=2 | 2050^a^/2457^a^ |

NA; not applicable.

^a^ Lost to follow-up.
