# Supplementary material for: Efficacy of single versus four repeated doses of praziquantel against Schistosoma mansoni infection in school-aged children from Côte d'Ivoire based on Kato-Katz and POC-CCA: An open-label, randomised controlled trial (RePST)
Source: PLoS Negl Trop Dis. 2020 Mar 20;14(3):e0008189. doi: 10.1371/journal.pntd.0008189 (PMC7112237; doi:10.1371/journal.pntd.0008189)
Supplement: S2 Table — Abbreviations: CR, cure rate; EPG, eggs per gram of stool; IRR, intensity reduction rate; NP, not possible; POC-CCA, point-of-care circulating cathodic antigen. a Number of infected children at baseline. b Measured 2 weeks post-treatment for one, two and three treatments and measured 4 weeks post-treatment for four treatments. c CR as calculated from the model based on the probability of being cured. d Median of the positives. e IRR based on the reduction in mean EPG as calculated from the model. f IRR based on the reduction in mean POC-CCA G-score as calculated manually. (DOCX) [file pntd.0008189.s007.docx]

**S2 Table. Cure and intensity reduction rates after one, two, three and four treatments with PZQ at two-week intervals in school-aged children infected with *S. mansoni* based on triplicate Kato-Katz thick smears from a single stool sample and single point-of-care circulating cathodic antigen (POC-CCA) urine cassette test.**

|  | **Standard treatment**  **group** | **Intense treatment**  **group** |  | |  |  |
| --- | --- | --- | --- | --- | --- | --- |
|  | First treatment  W0 | First treatment  W0 | Second treatment  W2 | | Third treatment  W4 | Fourth treatment  W6 |
| **Kato-Katz** | N=70 | N=83 | N=82 | | N=82 | N=78 |
| Infected children before treatment^a^ | 70 | 83 | 83 | | 83 | 83 |
| Cured children after treatment^b^ | 55 | 64 | 80 | | 76 | 69 |
| CR^c^ | 80.8%  (95% CI 69.6-88.6) | 80.0%  (95% CI 68.8-85.0) | 96.0%  (95% CI 89.2-98.6) | | 98.2%  (95% CI 91.4-99.7) | 86.0%  (95% CI 75.4-92.4) |
| Median EPG^d^ |  |  |  | |  |  |
| Before treatment | 172 | 128 | 128 | | 128 | 128 |
| After treatment^b^ | 8  *(out of 13 positive)* | 12  *(out of 18 positive)* | 8  *(out of 3 positive)* | | 8  *(one positive)* | 8  *(out of 11 positive)* |
| Arithmetic mean EPG |  |  |  | |  |  |
| Before treatment | 298.2 | 242.7 | 242.7 | | 242.7 | 242.7 |
| After treatment^b^ | 46.2 | 6.0 | 0.3 | | 0.1 | 3.2 |
| IRR^e^ | 95.6%  (95% CI 90.4-98.0) | 97.1%  (95% CI 94.1-98.6) | 99.9%  (95% CI 99.7-100.0) | | 100.0%  (95% CI 99.9-100.0) | 95.1%  (95% CI 85.1-98.4) |
| **POC-CCA (traces considered negative)** |  |  |  | |  |  |
| Infected children before treatment^a^ | 70 | 83 | 83 | | 83 | 83 |
| Cured children after treatment^b^ | 18 | 20 | 30 | | 29 | 29 |
| CR^c^ | 20.9%  (95% CI15.0-28.4) | 23.2%  (95% CI 16.6-31.3) | 44.4%  (95% CI 35.5-53.7) | | 47.7%  (95% CI 38.8-56.7) | 35.7%  (95% CI 26.4-46.1) |
| Median G-score^d^ |  |  | |  |  |  |
| Before treatment | 6 | 7 | 7 | | 7 | 7 |
| After treatment^b^ | 6 | 6 | 5 | | 5 | 6 |
| Arithmetic mean G-score |  |  |  | |  |  |
| Before treatment | 6.4 | 6.3 | 6.3 | | 6.3 | 6.3 |
| After treatment^b^ | 5.4 | 5.7 | 4.7 | | 4.2 | 4.6 |
| IRR^f^ | 15.6% | 9.5% | 25.4% | | 33.3% | 27.0% |

Abbreviations: CR, cure rate; EPG, eggs per gram of stool; IRR, intensity reduction rate; POC-CCA, point-of-care circulating cathodic antigen test.

^a^ Number of infected children at baseline

^b^ Measured 2 weeks post-treatment for one, two and three treatments and measured 4 weeks post-treatment for four treatments

^c^ CR as calculated from the model based on the probability of being cured

^d^ Median of the positives

^e^ IRR based on the reduction in mean EPG as calculated from the model

^f^ IRR based on the reduction in mean POC-CCA G-score as calculated manually
